# The association between cotinine-measured smoking intensity and sleep quality

**DOI:** 10.18332/tid/152221

**Published:** 2022-09-05

**Authors:** Supa Oh, Sinae Kim, Eunju Sung, Cheol-Hwan Kim, Jae-Heon Kang, Hocheol Shin, In Young Cho

**Affiliations:** 1 Department of Family Medicine, Kangbuk Samsung Hospital, Sungkyunkwan University School of Medicine, Seoul, Republic of Korea; 2 Division of Biostatistics, Department of R&D Management, Kangbuk Samsung Hospital, Sungkyunkwan University School of Medicine, Seoul, Republic of Korea

**Keywords:** cigarette smoking, cotinine, sleep quality

## Abstract

**INTRODUCTION:**

Cigarette smoking is suggested to be associated with sleep problems. This study evaluated the quantitative association between urinary cotinine-verified smoking intensity and sleep quality assessed by the Pittsburgh Sleep Quality Index (PSQI).

**METHODS:**

This was a cross-sectional study of 189970 participants from the Kangbuk Samsung Health Study recruited between 2016 and 2018. Logistic regression analysis adjusted for covariates was performed to estimate the association between urinary cotinine levels assessed by quartiles and poor sleep quality, defined as global PSQI score >5.

**RESULTS:**

The odds ratios (OR) and 95% confidence intervals (CI) for poor sleep quality comparing the highest urinary cotinine quartile to non-smokers were: 1.23 (95% CI: 1.16–1.30) for overall, 1.19 (95% CI: 1.12–1.26) for males, and 1.55 (95% CI: 1.29–1.87) for females. Among self-reported never smokers, cotinineverified smokers had higher odds for decreased sleep quality compared to cotinineverified never smokers with OR of 1.26 (95% CI: 1.08–1.46).

**CONCLUSIONS:**

Elevated urinary cotinine levels were associated with poor sleep quality in relatively young and middle-aged South Korean adults. Higher odds for poor sleep quality among cotinine-verified smokers who self-reported as never smokers also demonstrate the value of quantitative measurement of urinary cotinine. Prospective studies are warranted to clarify the cause-effect relationship between smoking and sleep quality.

## INTRODUCTION

Cigarette smoking is a serious public health problem. In spite of anti-smoking campaigns and public policies to discourage smoking, 1.1 billion smokers globally consumed 7.4 trillion cigarette-equivalents of tobacco in 2019^1^. In the same year, the smoking prevalence in men in Korea was 28.5%, which was the 4th highest rate among OECD countries^2^.

While the detrimental impacts of cigarette smoking on physical health have been well established, evidence supporting the effect of sleep quality on physical health is increasing. Poor sleep quality is associated with cardiovascular diseases^3^ and depression^4^. Hence, both abstinence from smoking and sleep quality are important for improving health outcomes.

Recently, a meta-analysis showed that smokers have a higher risk of developing sleep-related problems than non-smokers^5^. In a study using national survey data, patients with insomnia were more likely to smoke than those without insomnia^6^. However, previous studies mostly relied on selfreported smoking status. Only a few studies used biomarkers for differentiating smoking exposure, and those using biomarkers for evaluating smoking intensity reported inconsistent results. A Canadian national survey reported that increasing urinary cotinine levels were associated with higher odds of disturbed sleep quality^7^. In contrast, a German study using the Pittsburgh Sleep Quality Index (PSQI) showed no significant associations between smoking intensity measured by biological indicators (i.e. exhaled carbon dioxide, plasma cotinine) and sleep quality^8^.

Considering the underestimation of smoking in self-reports^9^, cotinine verification can provide more accurate information on smoking. Nicotine’s main metabolite, cotinine, is a widely used biomarker for smoking since it has a long half-life of 16 hours^10^. Urinary cotinine is non-invasive and highly correlated with serum cotinine^11^, and is suitable for assessing smoking status and intensity. Urinary cotinine is an objective measurement of smoking status with high sensitivity and specificity comparable to serum cotinine levels^12^.

Furthermore, most studies assessed only overall sleep quality or used self-developed questionnaires. By using a validated instrument, such as the PSQI^13^, overall poor sleep quality and a variety of sleep dimensions can be evaluated.

Therefore, this research aimed to analyze the quantitative association between cigarette smoking and sleep quality, by using urinary cotinine levels and a validated instrument for assessing sleep quality in a large population.

## METHODS

### Study design and population

Study subjects were participants of the Kangbuk Samsung Health Study, a cohort study of men and women aged ≥18 years who underwent comprehensive health screening examinations at Kangbuk Samsung Hospital Total Healthcare Centers in Seoul and Suwon, South Korea^14^. The Industrial Safety and Health Law in South Korea mandates annual or biennial health examinations for all employees; over 80% of the study participants were employees of companies or governmental organizations or their spouses, while the remaining received voluntary health checkups. The analysis included participants with visits between 2016 and 2018, and results for urinary cotinine and PSQI (n=202552). This study was approved by the Institutional Review Board of the Kangbuk Samsung Hospital (IRB No. KBSMC 2021-06-029), which waived the requirement for informed consent because we used pre-existing de-identified data obtained during regular health screening exams.

**Figure 1:**
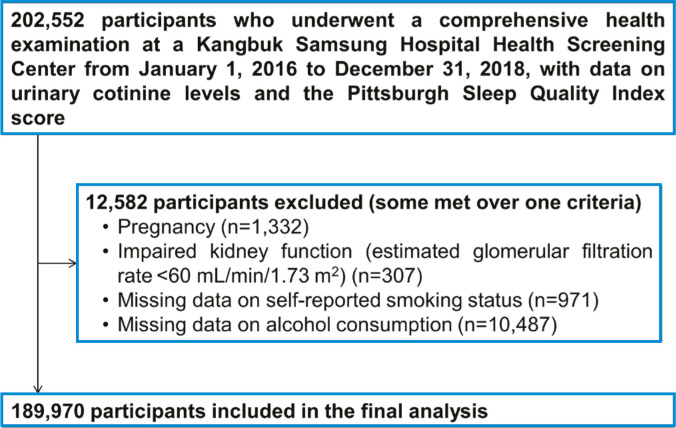
Flow chart of study participants

The exclusion criteria were as follows: pregnant women, due to accelerated metabolism of nicotine during pregnancy (n=1332)^15^; estimated glomerular filtration rate <60 mL/min/1.73m^2^, calculated using The Chronic Kidney Disease-Epidemiology Collaboration equation due to decreased metabolism of nicotine (n=307)^16^; and missing data on self-reported smoking status (n=971) and alcohol intake (n=10,487). Some individuals met more than one criterion for exclusion; as a result, 12582 were excluded and the remaining 189970 participants were included in the final analysis (Figure 1).

### Definition and variables

Data on sociodemographic characteristics, health status, and health behaviors were collected by standardized self-administered questionnaires and anthropometric measurements. The education level referred to the highest diploma received. Working status was categorized into daytime employment (working hours between 6 a.m. and 6 p.m.), non-daytime employment (working other hours), and unemployed. For history of chronic disease, participants were asked whether they had been diagnosed with one of the following conditions: hypertension, diabetes, dyslipidemia, asthma, chronic obstructive pulmonary disease, sleep apnea, heart disease, stroke, or cancer. For history of psychiatric disease, the following conditions were assessed: depressive disorder, narcolepsy, and insomnia. The use of psychiatric medications included antidepressants and other psychiatric medications, but did not include hypnotics, because it is a component of the PSQI. Depressive symptoms were assessed using the validated Korean version of the Center for Epidemiologic Studies Depression scale and a score of ≥16 was considered as clinically significant depressive symptoms^17^. Physical activity was assessed using the validated Korean version^18^ of the International Physical Activity Questionnaire short form. Alcohol intake was computed by the frequency and amount consumed per drinking session. Coffee consumption was assessed using the food frequency questionnaire for Koreans^19^.

Anthropometric parameters were measured by trained nurses. Obesity status was defined by body mass index (BMI, kg/m^2^) according to the criteria for obesity in Asians^20^.

Self-reported smoking questionnaires included information on current smoking status, smoking duration, and the number of cigarettes per day. Participants who smoked <100 cigarettes during their lifetime were classified as never smokers. Participants who smoked ≥100 cigarettes in their lifetime were categorized as: 1) current smokers or 2) former smokers, according to their current smoking status.

### Measurements

*Urinary cotinine*

Urinary cotinine was measured using the DRI Cotinine Assay (Microgenics Corp., Fremont, CA, USA) with a modular P800 (Roche Diagnostics, Tokyo, Japan). The participants were asked to refrain from eating or smoking for at least 10 hours before the comprehensive health screening examinations, at which the urine samples were collected. The cut-off used to distinguish cotinine-verified smokers from non-smokers was 50 ng/mL^10^. Cotinine-verified smokers were subdivided into quartiles, as in previous studies^7,11,21^.

*Pittsburgh Sleep Quality Index (PSQI)*

The PSQI is a validated self-reported questionnaire that evaluates sleep quality during the past month. This instrument consists of 19 items, grouped into seven components: subjective sleep quality, sleep latency, sleep duration, habitual sleep efficiency, sleep disturbance, use of sleep medication, and daytime dysfunction. Each component’s score ranges from 0 to 3; the seven component scores are summed for a global PSQI score, which ranges from 0 to 21. Higher scores indicate poor sleep quality; a global score >5 is considered as the range for identifying poor sleep quality^13^. The validated PSQI Korean version was used with a cut-off value of 5^22^. For each individual component, a score of ≥2 was used to identify clinically impaired domains, such as bad subjective sleep quality, sleep latency >30 minutes, sleep duration <6 hours, and sleep efficiency <75%^8,13^.

### Statistical analysis

To examine the quantitative relationship between cotinine-measured smoking intensity and sleep quality, urinary cotinine levels were categorized into five groups: the non-smoker group (<50 ng/mL), and smokers divided into quartiles distinctly for males and females (for males, Q1: 50–499 ng/mL; Q2: 500–1011 ng/mL; Q3: 1012–1598 ng/mL; and Q4: ≥1599 ng/mL; for females, Q1: 50–321 ng/mL; Q2: 322–685 ng/mL; Q3: 686–1211 ng/mL; and Q4 ≥1212 ng/mL).

The characteristics of the study population were summarized using descriptive statistics. To compare the participants’ characteristics according to urinary cotinine levels, the one-way ANOVA was used for continuous variables with a normal distribution. For continuous variables without a normal distribution such as alcohol consumption, the Kruskal-Wallis H test was performed. The chi-squared test was used for categorical variables.

Descriptive statistics were used to analyze smoking status according to self-report and urinary cotinine measurements. Kappa statistics were applied to calculate the degree of agreement between the two methods.

Multivariate logistic regression analyses were performed to evaluate the ORs and 95% CIs for poor sleep quality according to urinary cotinine level categories and self-reported smoking status. Models were initially adjusted for age and sex, and the multivariate model was further adjusted for education level, employment status, marital status, BMI, history of chronic disease, perceived health status, history of psychiatric disease, psychiatric medication use, depressive symptoms, physical activity, and alcohol consumption. The association between urinary cotinine level categories and sleep quality was also analyzed according to coffee consumption among participants with available data.

Sensitivity analyses were conducted with different cut-off points for PSQI and cotinine levels. For urinary cotinine, a higher cut-off of 100 ng/mL^23^ was used to distinguish smokers and non-smokers. For the PSQI score, a higher range >7 was used to distinguish poor sleep quality^24^.

All analyses were carried out using STATA version 16.1 (StataCorp LP, College station TX, USA). All reported p values were two-sided, and p<0.05 was considered statistically significant.

## RESULTS

The characteristics of the 189970 participants (108643 males and 81327 females) are presented in Supplementary file Table S1, according to urinary cotinine categories. The mean (standard deviation) age of the overall population was 33.83 (SD=6.02) years. Participant categories with higher cotinine levels were older, had higher unemployment levels, perceived self-health status poorly, reported more depressive symptoms, practiced higher levels of physical activity, and consumed more alcohol and coffee compared to the cotinine-verified non-smoker category. Cotinine-verified smokers showed higher levels of obesity than non-smokers, although obesity was lowest in the highest quartile among cotinineverified smokers. Characteristics according to sex are described in Supplementary file Tables S2 and S3.

Smoking status according to both urinary cotinine measurement and self-report are described in Table 1. The prevalence of smoking based on self-report and cotinine levels were 14.93% and 14.68%, respectively. The reliability between self-reported and cotinine-verified smoking status was strong overall (κ=0.896, standard error=0.003), and the reliability for males and females were κ=0.879 and κ=0.703, respectively. The percentage of misclassification, identified as cotinine-verified smokers who self-reported as never smokers, were 0.78%, 0.48%, and 0.92% among overall, male, and female participants, respectively.

**Table 1:** Smoking status by self-report and urinary cotinine verification

*Cotinine-verified smoking status*	*Self-reported smoking status*			*Total n (%)*
	*Never smoker n (%)*	*Former smoker n (%)*	*Current smoker n (%)*	
**Overall**				
Non-smoker	108813 (99.22)	49594 (95.49)	3679 (12.97)	162086 (85.32)
Smoker^a^	851 (0.78)	2343 (4.51)	24690 (87.03)	27884 (14.68)
**Male**				
Non-smoker	35096 (99.52)	44292 (95.74)	3487 (12.86)	82875 (76.28)
Smoker^a^	168 (0.48)	1973 (4.26)	23627 (87.14)	25768 (23.72)
**Female**				
Non-smoker	73717 (99.08)	5302 (93.48)	192 (15.30)	79211 (97.40)
Smoker^a^	683 (0.92)	370 (6.52)	1063 (84.70)	2116 (2.60)

The kappa values for agreement between self-reported and cotinine-verified smoking status among overall, male, and female subjects were 0.896, 0.879, and 0.703, respectively.

a Cotinine-verified smoker was defined by urinary cotinine level ≥50 ng/mL vs <50 ng/mL for non-smoker.

Overall, age and sex-adjusted ORs for poor sleep quality for increasing urinary cotinine quartiles were 1.26 (95% CI: 1.19–1.33), 1.31 (95% CI: 1.25–1.39), 1.42 (95% CI: 1.35–1.50), and 1.53 (95% CI: 1.45–1.61), respectively, compared to cotinine-verified non-smokers (p for trend <0.001) (Table 2). After further adjustment for covariates, increasing urinary cotinine quartiles were associated with increasing odds for poor sleep quality among overall, male, and female participants, with a stronger association among female participants (p for interaction=0.009). The multivariable-adjusted OR for poor sleep quality among overall participants was 1.011 (95% CI: 1.008–1.013) per 100 unit increase in urinary cotinine level.

**Table 2:** Odds ratios (95% confidence intervals) for poor sleep quality (global PSQI score >5) by cotinine-verified smoking status

*Smoking intensity*	*Age/sex adjusted OR^a^ (95% CI)*			*Multivariable-adjusted OR^b^ (95% CI)*		
	*Male*	*Female*	*Overall*	*Male*	*Female*	*Overall*
**Urinary cotinine level^c^**						
<50 (Ref.)	1	1	1	1	1	1
Quartile 1	1.23 (1.17–1.31)	1.39 (1.17–1.66)	1.26 (1.19–1.33)	1.11 (1.05–1.18)	1.09 (0.90–1.32)	1.11 (1.05–1.17)
Quartile 2	1.26 (1.19–1.34)	1.76 (1.48–2.09)	1.31 (1.25–1.39)	1.12 (1.05–1.19)	1.37 (1.13–1.65)	1.14 (1.08–1.21)
Quartile 3	1.37 (1.29–1.44)	1.88 (1.59–2.23)	1.42 (1.35–1.50)	1.17 (1.10–1.24)	1.38 (1.15–1.67)	1.19 (1.13–1.26)
Quartile 4	1.46 (1.38–1.54)	2.16 (1.82–2.57)	1.53 (1.45–1.61)	1.19 (1.12–1.26)	1.55 (1.29–1.87)	1.23 (1.16–1.30)
p for trend	<0.001	<0.001	<0.001	<0.001	<0.001	<0.001
Per 100 unit increase	1.019 (1.017–1.021)	1.049 (1.040–1.057)	1.021 (1.019–1.024)	1.009 (1.007–1.011)	1.026 (1.017–1.035)	1.011 (1.008–1.013)

PSQI: Pittsburgh Sleep Quality Index. p=0.009 for the overall interaction of sex in the multivariable-adjusted model for urinary cotinine levels.

a Sex was adjusted only for overall.

b Further adjusted for sex (only for overall), education level, employment status, marital status, body mass index, history of chronic disease, perceived health status, history of psychiatric disease, psychiatric medication use, depressive symptoms, physical activity, and alcohol consumption.

c Urinary cotinine levels ≥50 ng/mL were divided into quartiles, according to sex: Q1: 50–499 ng/mL; Q2: 500–1011 ng/mL; Q3: 1012–1598 ng/mL; Q4: ≥1599 ng/mL for male; and Q1: 50–321 ng/mL; Q2: 322–685 ng/mL; Q3: 686–1211 ng/mL; Q4: ≥1212 ng/mL for female.

Similar associations were observed between self-reported smoking and poor sleep quality (Table 3). For self-reported smoking status, the OR for current smokers was 1.21 (95% CI: 1.16–1.25) compared to never smokers after adjustment for covariates. Increasing smoking intensity showed a dose-response association with poor sleep quality, with OR of 1.10 (95% CI: 1.07–1.14), 1.20 (95% CI: 1.15–1.25), and 1.38 (95% CI: 1.22–1.56) for 1–10, 11–20, and >20 cigarettes per day, respectively.

**Table 3:** Odds ratios (95% confidence intervals) for poor sleep quality (global PSQI score >5) by self-reported smoking status

*Smoking intensity*	*Age (years)/sex adjusted OR^a^ (95% CI)*			*Multivariable-adjusted OR^b^ (95% CI)*		
	*Male*	*Female*	*Overall*	*Male*	*Female*	*Overall*
**Smoking status**						
Never smoker (Ref. )	1	1	1	1	1	1
Former smoker	1.05 (1.02–1.09)	1.21 (1.15–1.28)	1.10 (1.07–1.14)	1.06 (1.03–1.10)	1.06 (0.99–1.12)	1.06 (1.03–1.09)
Current smoker	1.39 (1.34–1.44)	2.10 (1.88–2.35)	1.48 (1.43–1.53)	1.20 (1.15–1.25)	1.37 (1.21–1.55)	1.21 (1.16–1.25)
p for trend	<0.001	<0.001	<0.001	<0.001	<0.001	<0.001
**Smoking amount** (cigarettes/day)						
0 (Ref.)	1	1	1	1	1	1
1–10	1.14 (1.10–1.17)	1.75 (1.63–1.88)	1.22 (1.19–1.26)	1.08 (1.04–1.12)	1.28 (1.18–1.38)	1.10 (1.07–1.14)
11–20	1.41 (1.36–1.46)	1.68 (1.32–2.15)	1.46 (1.41–1.52)	1.19 (1.14–1.24)	1.03 (0.79–1.35)	1.20 (1.15–1.25)
>20	1.92 (1.71–2.14)	2.26 (0.56–9.03)	1.99 (1.78–2.23)	1.37 (1.21–1.55)	1.18 (0.25–5.71)	1.38 (1.22–1.56)
p for trend	<0.001	<0.001	<0.001	<0.001	<0.001	<0.001

PSQI: Pittsburgh Sleep Quality Index.

a Sex was adjusted only for overall.

b Further adjusted for sex (only for overall), education level, employment status, marital status, body mass index, history of chronic disease, perceived health status, history of psychiatric disease, psychiatric medication use, depressive symptoms, physical activity, and alcohol consumption.

Analysis of the association between misclassified self-reported smoking exposure identified through urinary cotinine levels and poor sleep quality is shown in Table 4. Overall, compared to cotinine-verified non-smokers, the OR for poor sleep quality among cotinine-verified smokers was 1.17 (95% CI: 1.13–1.21) after adjustment for covariates. Among self-reported never smokers, the OR for poor sleep quality in cotinine-verified smokers, or assumedly misclassified as never smokers in self-report, was 1.26 (95% CI: 1.08–1.46) compared to cotinine-verified non-smokers, after adjustment for covariates.

**Table 4:** Analysis of misclassified participants by self-report identified through urinary cotinine levels and poor sleep quality (global PSQI score >5)

*Smoking status*	*Age (years)/sex adjusted OR^a^ (95% CI)*			*Multivariable-adjusted OR^b^ (95% CI)*		
	*Male*	*Female*	*Overall*	*Male*	*Female*	*Overall*
**Cotinine-verified smoking status^c^**						
Cotinine-verified non-smoker (Ref.)	1	1	1	1	1	1
Cotinine-verified smoker	1.33 (1.29–1.37)	1.78 (1.63–1.94)	1.37 (1.34–1.42)	1.15 (1.11–1.19)	1.34 (1.22–1.47)	1.17 (1.13–1.21)
**Among self-reported never smokers**						
Cotinine-verified nonsmoker (Ref.)	1	1	1	1	1	1
Cotinine-verified smoker	1.24 (0.88–1.75)	1.46 (1.25–1.70)	1.42 (1.23–1.63)	1.05 (0.72–1.53)	1.28 (1.09–1.52)	1.26 (1.08–1.46)

PSQI: Pittsburgh Sleep Quality Index.

a Sex was adjusted only for overall.

b Further adjusted for sex (only for overall), education level, employment status, marital status, body mass index, history of chronic disease, perceived health status, history of psychiatric disease, psychiatric medication use, depressive symptoms, physical activity, and alcohol consumption.

c Cotinine-verified smoker was defined by urinary cotinine level ≥50 ng/mL vs <50 ng/mL for non-smoker.

The percentage for impairment of each PSQI component tended to increase with increasing urinary cotinine quartile across all components, except for sleep disturbances among males (Supplementary file Table S4). For both males and females, the percentages with poor sleep quality and global PSQI scores both increased with increasing cotinine levels, with the lowest percentages and scores for non-smokers (p for trend <0.001). Among female smokers, 50% in the highest quartile of urinary cotinine level showed poor sleep quality, and mean global PSQI scores were >5 across all quartiles. The mean scores of each PSQI component are shown in Supplementary file Table S5.

In subgroup analysis of participants with available data on coffee consumption (n=114333), there was no significant difference in the association between urinary cotinine levels and sleep quality by amount of daily coffee intake (p for interaction=0.268) (Supplementary file Table S6).

In sensitivity analysis using urinary cotinine cut-off of 100 ng/mL, the percentage of cotinine-verified smokers slightly decreased (84.41% compared to 87.03% with cut-off of 50 ng/ml); the overall kappa value for agreement between self-reported and cotinine verified smoking status was 0.880 (Supplementary file Table S7). A similar doseresponse relationship between urinary cotinine quartiles and poor sleep quality was observed overall among combinations of different cut-off values for urinary cotinine levels (50 or 100 ng/mL) and PSQI scores (5 or 7) (Supplementary file Table S8).

## DISCUSSION

In this cross-sectional study including apparently healthy and young Korean adults, increasing urinary cotinine levels were associated with poor sleep quality in a dose-dependent trend. The association remained significant after adjustment for covariates including employment status and alcohol intake. The association was also consistent according to self-reported smoking status and increasing smoking intensity assessed by cigarettes per day.

Our study was the first to find a positive association between urinary cotinine levels and sleep quality using a validated questionnaire. A Canadian study reporting a positive association between increasing urinary cotinine levels and poor sleep quality used a self-reported questionnaire evaluating the total number of sleep problems, which was not a validated measure of sleep quality^7^. A German study, which used the PSQI, did not find a significant association between urinary cotinine levels and global PSQI scores; the only association found was for the PSQI component of sleep duration^8^.

The difference between self-reported and urinary cotinine-verified smoking prevalence observed in this study (14.94% vs 14.68%) was small compared to previous studies. In one systematic review, the average difference between self-reported and urinary cotinine measured smoking prevalence was 9.4%. In the Korea National Health and Nutritional Examination Survey (KNHANES) performed in 2016, the difference was 2.1%^25^. Moreover, the percentage of misclassified individuals (non-smokers based on self-reported data, but verified as active or passive smokers through urinary cotinine level ≥50 ng/mL) of 0.78% was lower than the 4.2% observed in the KNHANES data. This difference can be attributed to the characteristics of this study’s population. The majority of the participants were current employees of large companies. A strict non-smoking policy in the workplace, and incentives for smoking cessation provided by companies^26^ could reduce secondhand smoking, lowering the misclassification rate.

Nonetheless, misclassified never smokers (cotinine-verified smokers who self-reported as never smokers) showed higher odds for poor sleep quality compared to cotinine-verified self-reported never smokers. False reporting due to social pressure, secondhand exposure to smoking, or inappropriate cut-off levels for cotinine may cause misclassification. The association was stronger in females than males, possibly due to the higher impact of social desirability factor in Korean females than males^27^. The dose-response association between smoking intensity and poor sleep quality among females was also strong when examined by urinary cotinine quartiles, but less clear when examined by self-report of cigarettes/day, supporting the use of urinary cotinine measurements. Higher odds for poor sleep quality among cotinine-verified smokers, albeit self-reported never smokers, suggest the utility of quantitative measurement of urinary cotinine in alleged never smokers, who may be exposed to secondhand smoking or have provided a false report of smoking status. Our study was the first to show an association between urinary cotinine and sleep quality among misclassified never smokers, which were underrepresented in previous studies and may require evaluation and improvement of sleep health.

The dose-response association between urinary cotinine quartiles and poor sleep quality was stronger in females compared to males, which is consistent with previous studies^7,28^. In addition, a Korean study using a nationwide sample showed worse sleep quality for self-reported smokers versus non-smokers, with adjusted OR of 1.17 for males and 2.07 for females^29^. This discrepancy may be partially attributed to the difference in nicotine metabolism according to sex^30^. While the cytochrome P450 (CYP) 2A6 is a key enzyme for the metabolism of nicotine, female livers have more CYP2A6 protein and higher mRNA expression compared to male livers^31^. Furthermore, females could be influenced by nicotine-mediated reinforcement that contributes to nicotine addiction through estrogen modulation of dopamine receptors during menstrual cycles^32^. Estrogen can modulate the activity of enzymes that metabolize nicotine^33^ and accelerate glucuronide conjugation, an important pathway of nicotine metabolism^34^.

When examining individual PSQI components, the percentage of participants with impaired components increased with increasing urinary cotinine quartiles, except for sleep disturbances among males. The PSQI component of sleep disturbances includes questions on breathing difficulty, cough, or snoring, during sleep. Chronic diseases including sleep apnea could explain the discrepancy for sleep disturbances by urinary cotinine levels shown in males versus females. The percentage of sleep apnea was higher in males (1.88%) than in females (0.13%), while poor sleep quality rates and mean PSQI global scores were higher in females than in males, for both cotinine-verified non-smokers and smokers. These results are consistent with previous studies where females tend to report poor sleep quality more often than males^29^. However, the mechanisms for the differences by sex may encompass biological, psychological, and social factors^35^, and remain to be clarified.

The mechanisms for the association between urinary nicotine levels and poor sleep quality are yet unclear. Nicotine is reported to be associated with detrimental effects on sleep architecture. As nicotine activates the nicotinic receptor, the release of neurotransmitters such as acetylcholine, serotonin, and gamma-amino butyric acid, can affect the sleep–wake cycle^36^. In addition, nocturnal nicotine withdrawal during sleep is observed among smokers^37^. Conversely, poor sleep could lead to heavier smoking and interrupted smoking cessation, which is supported by a genetic study^38^. Sleep deprivation may also increase the desire to smoke for the purpose of reducing drowsiness^39^.

We performed subgroup analysis including information on coffee consumption because the data were available for only 60% of the participants, instead of adjusting for coffee consumption in our main analyses. Although participant categories with higher cotinine levels consumed more coffee compared to the cotinine-verified non-smoker category, which is consistent with previous studies^40^, the association between urinary cotinine quartiles and poor sleep quality remained consistent, with no significant interaction of coffee consumption. Few studies have investigated the effect of coffee consumption on the association between smoking and sleep disturbances, with conflicting results. Caffeine was not associated with polysomnography sleep variables among smokers in one study^41^; while in another study, coffee consumption confounded the association between smoking and insomnia^42^. While our study results suggest that the association between urinary cotinine and sleep quality are independent from coffee consumption, further studies are indicated to elucidate the simultaneous effect of cotinine and caffeine.

In our sensitivity analysis, different cut-off values for urinary cotinine level and PSQI score did not substantially affect the association between urinary cotinine and poor sleep quality. However, the overall kappa values for agreement between self-reported and cotinine verified smoking status decreased when the urinary cotinine cut-off was set to 100 ng/mL, indicating that the cut-off of 50 ng/mL has better reliability. Moreover, the higher cut-off of PSQI score of 7 was used in previous studies including transplant and breast cancer patients^24^ or primary insomnia and narcolepsy patients^43^. The high severity of symptoms in these patients could require higher cut-off values for PSQI scores, while our study included a relatively healthier population.

### Limitations

Our study has several limitations. First, some factors which may influence urinary cotinine levels were not considered. Information on the timing of the last cigarette smoked was unavailable, and changing values of cotinine levels during night may affect sleep quality. The Hawthorne effect might also influence the cotinine measurements as it was possible for the participants to modify their smoking habits several days ahead of their planned health screening examinations. However, considering that tobacco exposure patterns are relatively consistent over time, a single measurement of urinary cotinine may be a representative biomarker of average daily smoking exposure^44^. In addition, simultaneous analysis of self-reported smoking status and amount showed consistent associations between smoking and sleep quality found in our study. Second, urinary cotinine levels were analyzed without creatinine correction because data on urinary creatinine were unavailable. However, corrections can be counterproductive, as suggested in a study where urine cotinine-to-creatinine ratios were poorly correlated with serum cotinine in comparison to urine cotinine alone^45^, and many previous studies have used uncorrected urinary cotinine values^10^. Third, information on the use of nicotine replacement therapy or e-cigarettes was not collected, which could result in variations in cotinine levels. Fourth, although a validated instrument was used for assessing sleep quality, objective methods of measuring sleep quality such as actigraphy and polysomnography may strengthen the results. Additionally, due to the cross-sectional design of this study, the direction of causality between urinary cotinine and sleep quality could not be assessed. Finally, the study participants mainly consisted of young and middle-aged, relatively healthy Koreans. Hence, we cannot generalize our results to other ethnicities or populations with different characteristics.

Despite these limitations, to the best of our knowledge, this is the first study to demonstrate a dose-response association between smoking intensity confirmed by a quantitative nicotine metabolite and sleep quality using a validated questionnaire. Our findings support the use of urinary cotinine among participants with poor sleep quality for not only self-reported smokers, but also self-reported non-smokers for assessing smoke exposure. Clinicians involved in smoking cessation programs may also be encouraged to implement urinary cotinine measurements and the evaluation of sleep quality for improving both sleep and physical health.

## CONCLUSIONS

In this large cross-sectional study including young and middle-aged Korean adults, urinary cotinine-verified smoking intensity was associated with higher odds of poor sleep quality in a dose-response relationship, even after adjustment for covariates. The evaluation of sleep quality combined with smoking intensity may help identify individuals requiring interventions for not only improved physical health but also better sleep. Further research is needed to elucidate the underlying mechanisms and investigate the longitudinal association between cotinine levels and sleep quality to assess causal effects.

## Data Availability

The data supporting this research are available from the authors on reasonable request.
