# Supplementary material for: The association between cotinine-measured smoking intensity and sleep quality
Source: Tob Induc Dis. 2022 Sep 5;20:77. doi: 10.18332/tid/152221 (PMC9443078; doi:10.18332/tid/152221)
Supplement: Supplementary file 1 [file TID-20-77-s1.pdf]

Supplementary Materials

Supplementary Table S1. Baseline characteristics of the study population by urinary cotinine levels.

Characteristics	Total	Non-smokers[a]	Smokers, by urinary cotinine quartiles (ng/ml)[b]				p value	p for trend
			Q1	Q2	Q3	Q4		
Number	189,970 (100)	162,086 (85.32)	6,979 (3.75)	6975 (3.66)	6,960 (3.64)	6,970 (3.62)		
Sociodemographics								
Age (years)	33.83±6.02	33.61±6.00	34.42±5.78	34.85±6.00	35.35±6.06	35.84±6.07	<0.001	<0.001
Sex							<0.001	<0.001
Male	108,643 (57.19)	82,875 (51.13)	6,450 (92.42)	6,446 (92.42)	6,430 (92.39)	6,442 (92.42)		
Female	81,327 (42.81)	79,211 (48.87)	529 (7.58)	529 (7.58)	530 (7.61)	528 (7.58)		
Education ≥college graduate	160,712 (84.60)	137,407 (84.77)	6,158 (88.24)	6,000 (86.02)	5,713 (82.08)	5,434 (77.96)	<0.001	<0.001
Employment status							<0.001	<0.001
Employed	144,339 (75.98)	120,211 (74.17)	6,113 (87.59)	6,071 (87.04)	5,989 (86.05)	5,955 (85.43)		
Daytime shift	130,240 (68.56)	107,895 (66.57)	5,790 (82.96)	5,659 (81.13)	5,533 (79.50)	5,363 (76.94)		
Non-daytime shift	14,099 (7.42)	12,316 (7.60)	323 (4.63)	412 (5.91)	456 (6.55)	592 (8.49)		
Unemployed	44,444 (23.40)	40,899 (25.23)	825 (11.82)	848 (12.16)	923 (13.26)	949 (13.62)		

Marital status							<0.001	0.028
Married	154,557 (81.36)	131,932 (81.40)	5,751 (82.40)	5,687 (81.53)	5,631 (80.91)	5,556 (79.71)		
Other	33,856 (17.82)	28,872 (17.81)	1,159 (16.61)	1,234 (17.69)	1,253 (18.00)	1,338 (19.20)		
Health status								
BMI, kg/m^2							<0.001	<0.001
Underweight (<18.5)	8,747 (4.60)	8,248 (5.09)	81 (1.16)	107 (1.53)	121 (1.74)	190 (2.73)		
Normal (18.5 <23.0)	77,769 (40.94)	70,261 (43.35)	1,753 (25.12)	1,771 (25.39)	1,866 (26.81)	2,118 (30.39)		
Overweight (23.0 <25.0)	42,412 (22.33)	35,291 (21.77)	1,811 (25.95)	1,740 (24.95)	1,729 (24.84)	1,841 (26.41)		
Obesity (≥25.0)	60,994 (32.11)	48,245 (29.77)	3,331 (47.73)	3,355 (48.10)	3,243 (46.59)	2,820 (40.46)		
History of chronic disease (Yes)	49,385 (26.00)	39,410 (24.31)	2,487 (35.64)	2,564 (36.76)	2,505 (35.99)	2,419 (34.71)	<0.001	<0.001
Perceived health status							<0.001	<0.001
Very good	55,465 (29.20)	48,423 (29.87)	1,845 (26.44)	1,807 (25.91)	1,691 (24.30)	1,699 (24.38)		
Fair	116,296 (61.22)	98,773 (60.94)	4,292 (61.50)	4,371 (62.67)	4,448 (63.91)	4412 (63.30)		

Bad	17,807 (9.37)	14,560 (8.98)	824 (11.81)	780 (11.18)	804 (11.55)	839 (12.04)		
History of psychiatric disease (Yes)	6,804 (3.58)	5,732 (3.54)	256 (3.67)	260 (3.73)	276 (3.97)	280 (4.02)	0.080	0.004
Psychiatric medication use (Yes)	1,616 (0.85)	1,370 (0.85)	72 (1.03)	54 (0.77)	63 (0.91)	57 (0.82)	0.465	0.915
CES-D ≥16	19,450 (10.29)	16,609 (10.30)	676 (9.73)	662 (9.55)	707 (10.20)	796 (11.50)	0.002	0.183
Health behaviors								
Physical activity (IPAQ)							<0.001	<0.001
Low	94,959 (49.99)	81,528 (50.30)	3,241 (46.44)	3,284 (47.08)	3,371 (48.43)	3,535 (50.72)		
Moderate	66,760 (35.14)	56,795 (35.04)	2,680 (38.40)	2,590 (37.13)	2,466 (35.43)	2,229 (31.98)		
High	28,218 (14.85)	23,734 (14.64)	1,058 (15.16)	1,099 (15.76)	1,122 (16.12)	1,205 (17.29)		
Alcohol consumption (g/day)	6 (2-14)	4 (2-13)	14 (6-30)	14 (6-32)	14 (6-34)	14 (6-36)	<0.001	<0.001
Coffee consumption (cup/day)							<0.001	<0.001
<1	38,771 (20.41)	34,549 (21.32)	1,310 (18.77)	1,144 (16.4)	958 (13.76)	810 (11.62)		
1–2	50,853 (26.77)	43,964 (27.12)	1,843 (26.41)	1,784 (25.58)	1,714 (24.63)	1,548 (22.21)		
3–4	18,883 (9.94)	14,908 (9.20)	794 (11.38)	918 (13.16)	1,032 (14.83)	1,231 (17.66)		
≥5	5,826 (3.07)	4,475 (2.76)	240 (3.44)	260 (3.73)	361 (5.19)	490 (7.03)		

Unknown	75,637 (39.82)	64,190 (39.60)	2,792 (40.01)	2,869 (41.13)	2,895 (41.59)	2,891 (41.48)		
Self-reported smoking status							<0.001	<0.001
Never smoker	109,664 (57.73)	108,813 (67.13)	330 (4.73)	189 (2.71)	199 (2.86)	133 (1.91)		
Former smoker	51,937 (27.34)	49,594 (30.60)	1,160 (16.62)	505 (7.24)	359 (5.16)	319 (4.58)		
Current smoker	28,369 (14.93)	3,679 (2.27)	5,489 (78.65)	6,281 (90.05)	6,402 (91.98)	6,518 (93.52)		

BMI: body mass index. CES-D: Center for Epidemiologic Studies Depression Scale. IPAQ: International Physical Activity Questionnaire.

Data are presented as n (%), mean ± standard deviation, or median (interquartile range).

[a]Non-smokers were defined by urinary cotinine level <50 ng/ml.

[b]Urinary cotinine levels ≥50 ng/ml were divided into quartiles, according to sex: Q1, 50–499 ng/ml; Q2, 500–1,011 ng/ml; Q3, 1,012–1,598 ng/ml; Q4, ≥1,599 ng/ml for male; and Q1, 50–321 ng/ml; Q2, 322–685 ng/ml; Q3, 686–1,211 ng/ml; Q4, ≥1,212 ng/ml for female.

Supplementary Table S2. Baseline characteristics of males by urinary cotinine levels.

Characteristics	Total	Non-smokers[a]	Smokers, by urinary cotinine quartiles (ng/ml)[b]				P value	p for trend
			Q1	Q2	Q3	Q4		
Number	108,643	82,875	6,450	6,446	6,430	6,442		
Sociodemographics								
Age (years)	33.99±5.86	33.62±5.76	34.46±5.78	34.92±6.01	35.43±6.03	35.91±6.07	<0.001	<0.001
Education≥college graduate	96,315 (88.65)	74,434 (89.81)	5,775 (89.53)	5,625 (87.26)	5,375 (83.59)	5,106 (79.26)		
Employment status							<0.001	<0.001
Employed	97,181 (89.45)	74,326 (89.69)	5,775 (89.54)	5,739 (89.03)	5,678 (88.31)	5,663 (87.91)		
Daytime shift	90,076 (82.91)	68817 (83.04)	5,496 (85.21)	5,367 (83.26)	5,271 (81.98)	5,125 (79.56)		
Non-daytime shift	7,105 (6.54)	5,509 (6.65)	279 (4.33)	372 (5.77)	407 (6.33)	538 (8.35)		
Unemployed	10,836 (9.97)	8,121 (9.80)	637 (9.88)	651 (10.10)	708 (11.01)	719 (11.16)		
Marital status							<0.001	<0.001
Married	87,221 (80.28)	66,197 (79.88)	5,340 (82.79)	5,305 (82.30)	5,247 (81.60)	5,132 (79.66)		
Other	20,497 (18.87)	16,005 (19.31)	1,041 (16.14)	1,092 (16.94)	1,119 (17.40)	1,240 (19.25)		
Health status								

BMI (kg/m^2)							<0.001	0.630
Underweight (<18.5)	840 (0.77)	570 (0.69)	35 (0.54)	58 (0.90)	67 (1.04)	110 (1.71)		
Normal (18.5–<23.0)	27,396 (25.22)	21,141 (25.51)	1,430 (22.17)	1,449 (22.48)	1,561 (24.28)	1,815 (28.17)		
Overweight (23.0–<25.0)	30,768 (28.32)	23,928 (28.87)	1,740 (26.98)	1,666 (25.85)	1,662 (25.85)	1,772 (27.51)		
Obesity (≥25.0)	49,623 (45.68)	37,225 (44.92)	3,244 (50.29)	3,271 (50.74)	3,139 (48.82)	2,744 (42.60)		
History of chronic disease (Yes)	37,810 (34.80)	28,162 (33.98)	2,418 (37.49)	2,475 (38.40)	2,423 (37.68)	2,332 (36.20)	<0.001	<0.001
Perceived health status							<0.001	<0.001
Very good	33,654 (30.98)	27,087 (32.68)	1,707 (26.47)	1,681 (26.08)	1,586 (24.67)	1,593 (24.73)		
Fair	64,608 (59.47)	48,447 (58.46)	3,962 (61.43)	4,032 (62.55)	4,088 (63.58)	4,079 (63.32)		
Bad	10,152 (9.34)	7,179 (8.66)	766 (11.88)	717 (11.12)	740 (11.51)	750 (11.64)		
History of psychiatric disease (Yes)	2,994 (2.76)	2,148 (2.59)	206 (3.19)	204 (3.16)	219 (3.41)	217 (3.37)	<0.001	<0.001
Psychiatric medication use (Yes)	801 (0.74)	599 (0.72)	59 (0.91)	43 (0.67)	56 (0.87)	44 (0.68)	0.260	0.003
CES-D ≥16	8,107 (7.49)	5,778 (7.00)	554 (8.63)	544 (8.49)	572 (8.94)	659 (10.30)	<0.001	<0.001
Health behaviors								
Physical activity (IPAQ)							<0.001	<0.001

Low	47,428 (43.65)	35,315 (42.61)	2,909 (45.10)	2,962 (45.95)	3,040 (47.28)	3,202 (49.71)		
Moderate	42,858 (39.45)	33,413 (40.32)	2,548 (39.50)	2,447 (37.96)	2,344 (36.45)	2,106 (32.69)		
High	18,348 (16.89)	14,141 (17.06)	993 (15.40)	1,036 (16.07)	1,045 (16.25)	1,133 (17.59)		
Alcohol consumption (g/day)	10 (4–23)	9 (4–19)	14 (6–31)	14 (6–34)	14 (6–36)	15.5 (6 – 36)	<0.001	<0.001
Coffee consumption (cup/day)							<0.001	<0.001
<1	20,368 (18.75)	16,534 (19.95)	1,197 (18.56)	1,039 (16.12)	870 (13.53)	728 (11.30)		
1–2	28,207 (25.96)	21,868 (26.39)	1,701 (26.37)	1,646 (25.54)	1,579 (24.56)	1,413 (21.93)		
3–4	12,230 (11.26)	8,515 (10.27)	744 (11.53)	855 (13.26)	958 (14.90)	1,158 (17.98)		
≥5	3,149 (2.90)	1,926 (2.32)	217 (3.36)	231 (3.58)	325 (5.05)	450 (6.99)		
Unknown	44,689 (41.13)	34,032 (41.06)	2,591 (40.17)	2,675 (41.50)	2,698 (41.96)	2,693 (41.80)		
Self-reported smoking status							<0.001	<0.001
Never smoker	35,264 (32.46)	35,096 (42.35)	92 (1.43)	24 (0.37)	30 (0.47)	22 (0.34)		
Former smoker	46,265 (42.58)	44,292 (53.44)	1,060 (16.43)	413 (6.41)	273 (4.25)	227 (3.52)		

Current smoker	27,114 (24.96)	3,487 (4.21)	5,298 (82.14)	6,009 (93.22)	6,127 (95.29)	6,193 (96.13)

BMI: body mass index. CES-D: Center for Epidemiologic Studies Depression Scale. IPAQ: International Physical Activity Questionnaire.

Data are presented as mean ± standard deviation, median (interquartile range), or n (%).

[a]Non-smokers were defined by urinary cotinine level <50 ng/ml.

[b]Urinary cotinine levels ≥50 ng/ml were divided into quartiles: Q1, 50–499 ng/ml; Q2, 500–1,011 ng/ml; Q3, 1,012–1,598 ng/ml; Q4, ≥1,599 ng/ml.

Supplementary Table S3. Baseline characteristics of females by urinary cotinine levels.

Characteristics	Total	Non-smokers[a]	Smokers, by urinary cotinine quartiles (ng/ml)[b]				p value	p for trend
			Q1	Q2	Q3	Q4		
Number	81,327	79,211	529	529	530	528		
Sociodemographics								
Age (years)	33.61±6.23	33.60±6.24	33.94±5.70	33.96±5.88	34.47±6.27	34.9±6.01	<0.001	<0.001
Education ≥college graduate	64,397 (79.18)	62,973 (79.50)	383 (72.40)	375 (70.89)	338 (63.77)	328 (62.12)	<0.001	<0.001
Employment status							<0.001	0.883
Employed	47158(57.99)	45,885(57.92)	338(63.90)	332(62.76)	311(58.68)	292(55.31)		
Daytime shift	40,164 (49.39)	39,078 (49.33)	294 (55.58)	292 (55.20)	262 (49.43)	238 (45.08)		
Non-daytime shift	6,994 (8.60)	6,807 (8.59)	44 (8.32)	40 (7.56)	49 (9.25)	54 (10.23)		
Unemployed	33,608 (41.32)	32,778 (41.38)	188 (35.54)	197 (37.24)	215 (40.57)	230 (43.56)		
Marital status							<0.001	0.340
Married	67,336 (82.80)	65,735 (82.99)	411 (77.69)	382 (72.21)	384 (72.45)	424 (80.30)		
Other	13,359 (16.43)	12,867 (16.24)	118 (22.31)	142 (26.84)	134 (25.28)	98 (18.56)		
Health status								
BMI (kg/m^2)							<0.001	0.395
Underweight (<18.5)	7,907 (9.72)	7,678 (9.69)	46 (8.70)	49 (9.26)	54 (10.19)	80 (15.15)		
Normal (18.5–<23.0)	50,373 (61.94)	49,120 (62.01)	323 (61.06)	322 (60.87)	305 (57.55)	303 (57.39)		
Overweight (23.0–<25.0)	11,644 (14.32)	11,363 (14.35)	71 (13.42)	74 (13.99)	67 (12.64)	69 (13.07)		
Obesity (≥25.0)	11,371 (13.98)	11,020 (13.91)	87 (16.45)	84 (15.88)	104 (19.62)	76 (14.39)		

History of chronic disease (Yes)	11,575 (14.23)	11,248 (14.20)	69 (13.04)	89 (16.82)	82 (15.47)	87 (16.48)	0.169	0.043
Perceived health status							<0.001	<0.001
Very good	21,811 (26.82)	21,336 (26.94)	138 (26.09)	126 (23.82)	105 (19.81)	106 (20.08)		
Fair	51,688 (63.56)	50,326 (63.53)	330 (62.38)	339 (64.08)	360 (67.92)	333 (63.07)		
Bad	7,655 (9.41)	7,381 (9.32)	58 (10.96)	63 (11.91)	64 (12.08)	89 (16.86)		
History of psychiatric disease (Yes)	3,810 (4.68)	3,584 (4.52)	50 (9.45)	56 (10.59)	57 (10.75)	63 (11.93)	<0.001	<0.001
Psychiatric medication use (Yes)	815 (1.00)	771 (0.97)	13 (2.46)	11 (2.08)	7 (1.32)	13 (2.46)	<0.001	<0.001
CES-D ≥16	11,343 (14.05)	10,831 (13.77)	122 (23.33)	118 (22.48)	135 (25.52)	137 (26.20)	<0.001	<0.001
Health behaviors								
Physical activity (IPAQ)							0.001	0.121
Low	47,531 (58.44)	46,213 (58.34)	332 (62.76)	322 (60.87)	331 (62.45)	333 (63.07)		
Moderate	23,902 (29.39)	23,382 (29.52)	132 (24.95)	143 (27.03)	122 (23.02)	123 (23.30)		
High	9,870 (12.14)	9,593 (12.11)	65 (12.29)	63 (11.91)	77 (14.53)	72 (13.64)		
Alcohol consumption (g/day)	3 (1–6)	3 (1–6)	6 (3–16)	7 (3–19)	6 (2–17)	6 (2–20)	<0.001	<0.001
Coffee consumption (cup/day)							<0.001	<0.001
<1	18,403 (22.63)	18,015 (22.74)	113 (21.36)	105 (19.85)	88 (16.60)	82 (15.53)		
1–2	22,646 (27.85)	22,096 (27.90)	142 (26.84)	138 (26.09)	135 (25.47)	135 (25.57)		
3–4	6,653 (8.18)	6,393 (8.07)	50 (9.45)	63 (11.91)	74 (13.96)	73 (13.83)		
≥5	2,677 (3.29)	2,549 (3.22)	23 (4.35)	29 (5.48)	36 (6.79)	40 (7.58)		
Unknown	30,948 (38.05)	30,158 (38.07)	201 (38.00)	194 (36.67)	197 (37.17)	198 (37.50)		

Self-reported smoking status							<0.001	<0.001
Never smoker	74,400 (91.48)	73,717 (93.06)	238 (44.99)	165 (31.19)	169 (31.89)	111 (21.02)		
Former smoker	5,672 (6.97)	5,302 (6.69)	100 (18.90)	92 (17.39)	86 (16.23)	92 (17.42)		
Current smoker	1,255 (1.54)	192 (0.24)	191 (36.11)	272 (51.42)	275 (51.89)	325 (61.55)		

BMI: body mass index. CES-D: Center for Epidemiologic Studies Depression Scale. IPAQ: International Physical Activity Questionnaire.

Data are presented as mean±standard deviation, median (interquartile range), or n (%).

[a]Non-smokers were defined by urinary cotinine level <50 ng/ml.

[b]Urinary cotinine levels ≥50 ng/ml were divided into quartiles: Q1, 50–321 ng/ml; Q2, 322–685 ng/ml; Q3, 686–1,211 ng/ml; Q4, ≥1,212 ng/ml.

Supplementary Table S4. Numbers and percentages with impaired components (scores ≥2) and global scores of the Pittsburgh Sleep Quality Index (PSQI) by urinary cotinine levels.

PSQI component	Total	Non-smokers[a]	Smokers, by urinary cotinine quartiles (ng/ml)[b]				p value	p for trend
			Q1	Q2	Q3	Q4		
Male								
Subjective sleep quality score≥2	19,156 (17.63)	14,222 (17.16)	1,178 (18.26)	1,215 (18.85)	1,261 (19.61)	1,280 (19.87)	<0.001	<0.001
Sleep latency score≥2	14,811 (13.63)	10,204 (12.31)	1,030 (15.97)	1,070 (16.6)	1,242 (19.32)	1,265 (19.64)	<0.001	<0.001
Sleep duration score≥2	27,243 (25.08)	19,964 (24.09)	1,684 (26.11)	1,775 (27.54)	1,881 (29.25)	1,939 (30.10)	<0.001	<0.001
Habitual sleep efficiency score≥2	4,522 (4.16)	3,316 (4.00)	288 (4.47)	302 (4.69)	298 (4.63)	318 (4.94)	<0.001	<0.001
Sleep disturbances score≥2	1,416 (1.30)	1,063 (1.28)	91 (1.41)	77 (1.19)	100 (1.56)	85 (1.32)	0.327	0.307
Use of sleep medication score≥2	662 (0.61)	467 (0.56)	47 (0.73)	44 (0.68)	51 (0.79)	53 (0.82)	0.010	<0.001
Daytime dysfunction score≥2	12,259 (11.28)	9,250 (11.16)	756 (11.72)	702 (10.89)	757 (11.77)	794 (12.33)	0.017	0.006
Poor sleep quality (global score>5)	26,894 (24.75)	19,455 (23.48)	1,768 (27.41)	1,796 (27.86)	1,891 (29.41)	1,984 (30.80)	<0.001	<0.001
PSQI global score	4.23±2.38	4.15±2.34	4.39±2.45	4.43±2.42	4.55±2.49	4.63±2.52	<0.001	<0.001
Female								
Subjective sleep quality score≥2	21,475	20,741	166 (31.38)	179 (33.84)	191 (36.04)	198 (37.50)	<0.001	<0.001

	(26.41)	(26.18)						
Sleep latency score≥2	21,240 (26.12)	20,364 (25.71)	201 (38.00)	210 (39.70)	220 (41.51)	245 (46.40)	<0.001	<0.001
Sleep duration score≥2	15,576 (19.15)	14,996 (18.93)	118 (22.31)	140 (26.47)	162 (30.57)	160 (30.30)	<0.001	<0.001
Habitual sleep efficiency score≥2	6,941 (8.53)	6,728 (8.49)	55 (10.40)	46 (8.70)	50 (9.43)	62 (11.74)	0.040	<0.001
Sleep disturbances score≥2	3,207 (3.94)	3,090 (3.90)	23 (4.35)	22 (4.16)	38 (7.17)	34 (6.44)	<0.001	<0.001
Use of sleep medication score≥2	908 (1.12)	837 (1.06)	10 (1.89)	23 (4.35)	15 (2.83)	23 (4.36)	<0.001	<0.001
Daytime dysfunction score≥2	8,669 (10.66)	8,382 (10.58)	61 (11.53)	80 (15.12)	74 (13.96)	72 (13.64)	<0.001	<0.001
Poor sleep quality (global score>5)	26,172 (32.18)	25,215 (31.83)	208 (39.32)	238 (44.99)	247 (46.60)	264 (50.00)	<0.001	<0.001
PSQI global score	4.76±2.77	4.74±2.75	5.40±3.01	5.65±3.02	5.85±3.11	6.01±3.29	<0.001	<0.001

Data are presented as n (%) or mean ± standard deviation.

[a]Non-smokers were defined by urinary cotinine level <50 ng/ml.

[b]Urinary cotinine levels ≥50 ng/ml were divided into quartiles, according to sex: Q1, 50–499 ng/ml; Q2, 500–1,011 ng/ml; Q3, 1,012–1,598 ng/ml; Q4, ≥1,599 ng/ml for male; and Q1, 50–321 ng/ml; Q2, 322–685 ng/ml; Q3, 686–1,211 ng/ml; Q4, ≥1,212 ng/ml for female.

Supplementary Table S5. Components and global scores of the Pittsburgh Sleep Quality Index (PSQI) by urinary cotinine levels.

PSQI component	Total	Non-smokers[a]	Smokers, by urinary cotinine quartiles (ng/ml)[b]				p value	p for trend
			Q1	Q2	Q3	Q4		
Male								
Subjective sleep quality score	1.07±0.57	1.06±0.56	1.09±0.57	1.09±0.57	1.11±0.57	1.12±0.58	<0.001	<0.001
Sleep latency score	0.66±0.78	0.62±0.76	0.73±0.81	0.74±0.81	0.80±0.85	0.83±0.85	<0.001	<0.001
Sleep duration score	0.99±0.82	0.97±0.81	1.00±0.84	1.04±0.84	1.07±0.85	1.08±0.87	<0.001	<0.001
Habitual sleep efficiency score	0.21±0.57	0.20±0.55	0.23±0.60	0.23±0.60	0.24±0.59	0.25±0.62	<0.001	<0.001
Sleep disturbances score	0.64±0.51	0.63±0.51	0.65±0.51	0.64±0.51	0.63±0.51	0.63±0.51	0.023	0.558
Use of sleep medication score	0.02±0.22	0.02±0.21	0.03±0.23	0.02±0.23	0.03±0.24	0.03±0.25	0.015	0.001
Daytime dysfunction score	0.65±0.70	0.64±0.70	0.66±0.71	0.66±0.69	0.67±0.71	0.69±0.71	<0.001	<0.001
PSQI global score	4.23±2.38	4.15±2.34	4.39±2.45	4.43±2.42	4.55±2.49	4.63±2.52	<0.001	<0.001
Female								
Subjective sleep quality score	1.21±0.60	1.21±0.60	1.28±0.66	1.30±0.63	1.36±0.66	1.40±0.67	<0.001	<0.001
Sleep latency score	0.97±0.92	0.96±0.92	1.24±1.01	1.31±1.00	1.31±1.06	1.43±1.07	<0.001	<0.001
Sleep duration score	0.76±0.87	0.75±0.87	0.88±0.94	0.94±0.93	1.02±0.95	1.06±0.99	<0.001	<0.001
Habitual sleep efficiency score	0.35±0.74	0.35±0.74	0.43±0.82	0.37±0.75	0.43±0.79	0.44±0.83	<0.001	<0.001
Sleep disturbances score	0.81±0.48	0.81±0.48	0.84±0.48	0.87±0.45	0.89±0.50	0.86±0.51	<0.001	<0.001
Use of sleep medication score	0.04±0.30	0.04±0.29	0.09±0.43	0.14±0.57	0.10±0.44	0.15±0.57	<0.001	<0.001
Daytime dysfunction score	0.61±0.70	0.61±0.70	0.63±0.72	0.72±0.74	0.74±0.73	0.68±0.73	<0.001	<0.001
PSQI global score	4.76±2.77	4.74±2.75	5.40±3.01	5.65±3.02	5.85±3.11	6.01±3.29	<0.001	<0.001

Data are presented as mean ± standard deviation.

[a]Non-smokers were defined by urinary cotinine level <50 ng/ml.

[b]Urinary cotinine levels ≥50 ng/ml were divided into quartiles, according to sex: Q1, 50–499 ng/ml; Q2, 500–1,011 ng/ml; Q3, 1,012–1,598 ng/ml; Q4, ≥1,599 ng/ml for male; and Q1, 50–321 ng/ml; Q2, 322–685 ng/ml; Q3, 686–1,211 ng/ml; Q4, ≥1,212 ng/ml for female.

Supplementary Table S6. Subgroup analysis of poor sleep quality and urinary cotinine levels among participants with data on daily coffee consumption.

Urinary cotinine level[a]	OR (95% CI) for poor sleep quality by amount of daily coffee consumption				p for interaction
	<1 cup/day (n=38,771)	1–2 cups/day (n=50,853)	3–4 cups/day (n=18,883)	≥5 cups/day (n=5,826)	
Sex/age adjusted					
<50	1 (reference)	1 (reference)	1 (reference)	1 (reference)	
Quartile 1	1.20 (1.06–1.36)	1.27 (1.15–1.42)	1.32 (1.13–1.54)	1.33 (1.01–1.75)	
Quartile 2	1.30 (1.14–1.48)	1.47 (1.32–1.63)	1.37 (1.19–1.58)	1.22 (0.93–1.60)	0.256
Quartile 3	1.58 (1.38–1.81)	1.33 (1.19–1.48)	1.32 (1.15–1.52)	1.72 (1.37–2.17)	
Quartile 4	1.55 (1.34–1.80)	1.57 (1.40–1.75)	1.47 (1.29–1.66)	1.45 (1.18–1.78)	
p for trend	<0.001	<0.001	<0.001	<0.001	
Per 100 unit increase in cotinine level	1.026 (1.020–1.032)	1.023 (1.018–1.027)	1.017 (1.012–1.022)	1.020 (1.012–-1.028)	0.311
Multivariable-adjusted[b]					
<50	1 (reference)	1 (reference)	1 (reference)	1 (reference)	
Quartile 1	1.05 (0.92–1.20)	1.10(0.98–1.23)	1.18 (1.00–1.39)	1.26 (0.94–1.68)	
Quartile 2	1.09 (0.95–1.26)	1.26 (1.13–1.41)	1.22 (1.04–1.42)	1.01 (0.75–1.35)	0.268
Quartile 3	1.36 (1.17–1.57)	1.12 (1.00–1.26)	1.13 (0.97–1.30)	1.45 (1.14–1.86)	
Quartile 4	1.17 (1.00–1.37)	1.24 (1.10–1.40)	1.19 (1.04–1.36)	1.16 (0.93–1.45)	
p for trend	<0.001	<0.001	0.001	0.021	

Per 100 unit increase in cotinine level	1.012 (1.006–1.018)	1.011 (1.006–1.016)	1.007 (1.002–1.013)	1.010 (1.001–1.019)	0.932

CI: confidence interval. OR: odds ratio.

[a]Urinary cotinine levels ≥50 ng/ml were divided into quartiles, according to sex: Q1, 50–499 ng/ml; Q2, 500–1,011 ng/ml; Q3, 1,012–1,598 ng/ml; Q4, ≥1,599 ng/ml for male; and Q1, 50–321 ng/ml; Q2, 322–685 ng/ml; Q3, 686–1,211 ng/ml; Q4, ≥1,212 ng/ml for female.

[b]Further adjusted for education, employment status, marital status, body mass index, history of chronic disease, perceived health status, history of psychiatric disease, psychiatric medication use, depressive symptoms, regular physical activity, and alcohol consumption.

Supplementary Table S7. Smoking status by self-report and urinary cotinine verification with cut-off value of 100 ng/ml

Cotinine-verified smoking status	Self-reported smoking status			Total
	Never smoker	Former Smoker	Current smoker	
Overall				
Non-smoker	108,948 (99.35)	50,001 (96.27)	4,424 (15.59)	163,373 (86.00)
Smoker[a]	716 (0.65)	1,936 (3.73)	23,945 (84.41)	26,597 (14.00)
Male				
Non-smoker	35,129 (99.62)	44,663 (96.54)	4,188 (15.45)	83,980 (77.30)
Smoker[a]	135 (0.38)	1,602 (3.46)	22,926 (84.55)	24,663 (22.70)
Female				
Non-smoker	73,819 (99.22)	5,538 (94.11)	236 (18.80)	79,393 (97.62)
Smoker[a]	581 (0.78)	334 (5.89)	1,019 (81.20)	1,934 (2.38)

Data are presented as n (%).

The kappa values for agreement between self-reported and cotinine-verified smoking status among overall, male, and female subjects were 0.880, 0.857, and 0.708, respectively.

[a]Cotinine-verified smoker was defined by urinary cotinine level ≥100 ng/ml vs. <100 ng/ml for non-smoker.

Supplementary Table S8. Odds ratios and 95% confidence intervals for poor sleep quality by cotinine-verified smoking status with different cut-offs.

Smoking intensity	Age/sex adjusted OR[a] (95% CI)			Multivariable-adjusted OR[b] (95% CI)		
	Male	Female	Overall	Male	Female	Overall
Urinary cotinine <50 & PSQI > 7						
Urinary cotinine level[c]						
<50	1 (reference)	1 (reference)	1 (reference)	1 (reference)	1 (reference)	1 (reference)
Quartile 1	1.23 (1.13–1.34)	1.45 (1.17–1.79)	1.27 (1.18–1.38)	1.08 (0.99–1.19)	1.08 (0.85–1.36)	1.08 (1.00–1.18)
Quartile 2	1.26 (1.16–1.37)	1.75 (1.43–2.13)	1.34 (1.24–1.44)	1.08 (0.99–1.18)	1.31 (1.05–1.64)	1.12 (1.03–1.21)
Quartile 3	1.41 (1.30–1.53)	2.15 (1.78–2.60)	1.52 (1.41–1.63)	1.15 (1.06–1.26)	1.52 (1.23–1.88)	1.21 (1.12–1.31)
Quartile 4	1.56 (1.44–1.69)	2.33 (1.93–2.82)	1.67 (1.55–1.79)	1.19 (1.10–1.30)	1.59 (1.29–1.96)	1.27 (1.18–1.37)
p for trend	<0.001	<0.001	<0.001	<0.001	<0.001	<0.001
Urinary cotinine <100 & PSQI > 5						
Urinary cotinine level[d]						
<100	1 (reference)	1 (reference)	1 (reference)	1 (reference)	1 (reference)	1 (reference)
Quartile 1	1.22 (1.15–1.29)	1.73 (1.45–2.07)	1.27 (1.20–1.34)	1.10 (1.03–1.17)	1.32 (1.09–1.61)	1.11 (1.05–1.18)
Quartile 2	1.28 (1.21–1.35)	1.62 (1.35–1.94)	1.32 (1.25–1.39)	1.13 (1.06–1.20)	1.26 (1.04–1.53)	1.14 (1.08–1.21)
Quartile 3	1.35 (1.28–1.43)	2.04 (1.71–2.44)	1.42 (1.34–1.49)	1.15 (1.08–1.22)	1.48 (1.22–1.80)	1.18 (1.12–1.26)
Quartile 4	1.47 (1.39–1.56)	2.14 (1.79–2.56)	1.54 (1.46–1.62)	1.20 (1.12–1.27)	1.54 (1.26–1.87)	1.24 (1.17–1.31)

p for trend	<0.001	<0.001	<0.001	<0.001	<0.001	<0.001
Urinary cotinine <100 & PSQI > 7						
Urinary cotinine level[d]						
<100	1 (reference)	1 (reference)	1 (reference)	1 (reference)	1 (reference)	1 (reference)
Quartile 1	1.21 (1.11–1.32)	1.70 (1.38–2.10)	1.28 (1.19–1.39)	1.06 (0.97–1.17)	1.23 (0.98–1.56)	1.08 (0.99–1.18)
Quartile 2	1.29 (1.19–1.40)	1.68 (1.36–2.08)	1.35 (1.25–1.46)	1.10 (1.00–1.20)	1.27 (1.01–1.60)	1.13 (1.04–1.23)
Quartile 3	1.42 (1.31–1.54)	2.28 (1.87–2.78)	1.53 (1.42–1.65)	1.16 (1.06–1.26)	1.57 (1.26–1.96)	1.22 (1.12–1.32)
Quartile 4	1.56 (1.44–1.69)	2.23 (1.82–2.71)	1.65 (1.54–1.78)	1.19 (1.09–1.30)	1.52 (1.22–1.90)	1.26 (1.17–1.37)
p for trend	<0.001	<0.001	<0.001	<0.001	<0.001	<0.001

CI: confidence interval. OR: odds ratio. PSQI: Pittsburgh Sleep Quality Index.

[a]Sex was adjusted only for overall.

[b]Further adjusted for sex (only for overall), education, employment status, marital status, body mass index, history of chronic disease, perceived health status, history of psychiatric disease, psychiatric medication use, depressive symptoms, physical activity, and alcohol consumption.

[c]Urinary cotinine levels ≥50 ng/ml were divided into quartiles, according to sex: Q1, 50–499 ng/ml; Q2, 500–1,011 ng/ml; Q3, 1,012–1,598 ng/ml; Q4, ≥1,599 ng/ml for male; and Q1, 50–321 ng/ml; Q2, 322–685 ng/ml; Q3, 686–1,211 ng/ml; Q4, ≥1,212 ng/ml for female.

[d]Urinary cotinine levels ≥100 ng/ml were divided into quartiles, according to sex: Q1, 100–566 ng/ml; Q2, 567–1,057 ng/ml; Q3, 1,058–1,633 ng/ml; Q4, ≥1,634 ng/ml for male; and Q1, 100–402 ng/ml; Q2, 403–757 ng/ml; Q3, 758–1,279 ng/ml; Q4, ≥1,280 ng/ml for female.

© 2022 Oh S. et al.
